# Erratum

**DOI:** 10.1155/1994/51730

**Published:** 1994


					ERRATUM
In volume 7 number 1, page 13-14, ofHPB Surgery, the co-authors to the Invited Commentary
for the paper titled Histopathological Determinants of Survival in Resected Cases of
Pancreas Cancer should have appeared as follows:
J. L. AGUAYO-ALBASIMI and R. C. N. WILLIAMSON
The publishers would like to sincerely apologise for this error.

				

